# Durable Progression-Free and Treatment-Free Survival After Nivolumab Plus Ipilimumab Therapy in Metastatic Renal Cell Carcinoma: A Real-World Study with a 5-Year Minimum Follow-Up

**DOI:** 10.3390/cancers18081315

**Published:** 2026-04-21

**Authors:** Hiroaki Ikoma, Shuzo Hamamoto, Yoshihiko Tasaki, Misato Tomita, Kengo Kawase, Hiroko Suzuki, Yusuke Noda, Masayuki Usami, Yohei Tsubouchi, Ryuga Kato, Takuya Sakata, Yoshihisa Mimura, Toshiharu Morikawa, Takashi Nagai, Rei Unno, Toshiki Etani, Taku Naiki, Yosuke Sugiyama, Takahiro Yasui

**Affiliations:** 1 Department of Nephro–Urology, Nagoya City University Graduate School of Medical Sciences, 1 Kawasumi, Mizuho–cho, Mizuho–ku, Nagoya, Aichi 467–8601, Japan; urokoma@med.nagoya-cu.ac.jp (H.I.); t-mrkw@med.nagoya-cu.ac.jp (T.M.); uroetani@med.nagoya-cu.ac.jp (T.E.);; 2 Department of Clinical Pharmaceutics, Nagoya City University Graduate School of Medical Sciences, 1 Kawasumi, Mizuho–cho, Mizuho–ku, Nagoya, Aichi 467–8601, Japan; 3 Department of Urology, Kainan Hospital, 396 Minamihonda, Maegasu–cho, Yatomi, Aichi 498–8502, Japan; 4 Department of Urology, Anjo Kosei Hospital, 28 Higashihirokute, Anjo–cho, Anjo, Aichi 446–8602, Japan; 5 Department of Urology, Toyota Kosei Hospital, 1–500 Ibobara, Josui–cho, Toyota, Aichi 470–0396, Japan; 6 Department of Urology, Konan Kosei Hospital, 137 Omatsubara, Takaya–cho, Konan, Aichi 483–8704, Japan; 7 Department of Urology, Nagoya Tokushukai General Hospital, 2–52 Takakuraji–cho Kita, Kasugai, Aichi 487–0013, Japan; 8 Department of Urology, Gamagori City General Hospital, 1–1 Goshonishi–machi, Goi–cho, Gamagori, Aichi 443–8501, Japan

**Keywords:** metastatic renal cell carcinoma, immune checkpoint inhibitor, adverse event, treatment-free survival

## Abstract

Background/Objectives: Nivolumab plus ipilimumab (IO–IO) provides durable clinical benefit in metastatic renal cell carcinoma (mRCC), yet long-term real-world data focusing on progression-free and treatment-free (PF–TF) survival remain limited. This study aimed to evaluate the long-term outcomes of IO–IO with a particular focus on the frequency and clinical characteristics of PF–TF. Methods: We retrospectively analyzed 63 patients with mRCC treated with first-line IO–IO across eight institutions with a minimum potential follow-up of five years. Progression-free survival (PFS), PFS2, and overall survival (OS) were assessed. PF–TF was defined as absence of disease progression and any cancer-directed therapy at the five-year landmark. Clinical and treatment-related factors were compared between patients with and without PF–TF. Results: The median PFS, PFS2, and OS were 7.5 (95% confidence interval [CI], 5.1–13.3), 26.2 (95% CI, 13.6–46.6), and 47.4 months (95% CI, 29.3–not reached), respectively. At 5 years, 11 patients (17%) achieved PF–TF. Baseline characteristics, IMDC risk classification, and peripheral blood biomarkers were not predictive of PF–TF. PF–TF was associated with the absence of bone metastases, presence of lymph node metastases, and occurrence of immune-related adverse events (irAEs), as well as the delayed onset of irAEs. No PF–TF patients required corticosteroid pulse therapy, and durable PF–TF was observed even after early treatment discontinuation due to adverse events. Conclusions: IO–IO demonstrated sustained long-term efficacy in real-world practice, with a subset achieving durable PF–TF. These findings highlight IO–IO as a strategy capable of providing long-term disease control with reduced treatment burden in selected patients with mRCC.

## Simple Summary

Immune checkpoint inhibitor combination therapy with nivolumab plus ipilimumab can provide long-lasting disease control in patients with metastatic renal cell carcinoma, yet long-term real-world data remain limited. In this multicenter study with a minimum follow-up of 5 years, we found that a subset of patients achieved durable progression-free and treatment-free survival, even after early treatment discontinuation. Importantly, this long-term benefit was not predicted by conventional risk factors or blood tests but was associated with metastatic patterns, the development of irAEs, and delayed irAEs onset. These findings highlight the ability of this therapy to induce sustained immune-mediated tumor control and inform clinical decision making when selecting first-line therapy.

## 1. Introduction

Immune checkpoint inhibitor (ICI)-based combination therapy has been established as the standard first-line treatment for patients with metastatic renal cell carcinoma (mRCC) [1,2]. Nivolumab plus ipilimumab (IO–IO), the first ICI-based combination to become available, demonstrated a significant overall survival (OS) benefit compared with sunitinib in the phase III randomized CheckMate 214 trial [3,4]. Extended follow-up analyses have consistently shown a persistent “long tail” pattern in the survival curve [5]. Moreover, in the clinical trial, long-term follow-up reports have highlighted that a subset of patients achieves sustained disease control even after treatment discontinuation, underscoring durable progression-free and treatment-free (PF–TF) survival as a key clinical value of IO–IO therapy [6].

Furthermore, the survival benefits of first-line ICI plus tyrosine kinase inhibitor (IO–TKI) therapies have been widely reported, and several regimens have been established as treatment options [7,8,9,10]. However, these regimens are generally administered as continuous therapy, and durable disease control without ongoing therapy has not been well characterized. In this context, understanding the long-term clinical value of IO–IO therapy, particularly with respect to progression-free survival (PFS) and treatment-free disease control, is of increasing clinical importance.

Although multiple real-world studies have reported the outcomes of IO–IO therapy, the majority have relatively short follow-up periods, typically around 2–3 years [6,11,12,13,14]. As a result, these studies may be insufficient to provide mature OS estimates or to comprehensively describe long-term treatment trajectories. In addition, although PF–TF survival is considered a fundamental clinical benefit of IO–IO therapy, real-world evaluations using clearly defined endpoints that incorporate treatment-free intervals and long-term disease control have been limited, with only a few recent reports addressing these outcomes [15]. Therefore, reassessing the long-term clinical value of IO–IO therapy in routine practice based on follow-up beyond 5 years remains clinically meaningful.

In this retrospective study, we analyzed patients with mRCC treated with IO–IO and reported long-term clinical outcomes with a minimum follow-up of 5 years. We aimed to determine the frequency and clinical characteristics of PF–TF survival, a distinctive long-term outcome of IO–IO therapy. We also analyzed longitudinal blood test data using landmark analyses and evaluated treatment trajectories to better characterize long-term clinical courses in real-world practice.

## 2. Materials and Methods

### 2.1. Patient Enrollment

This study was approved by the Ethics Review Board of the Nagoya City University Graduate School of Medical Sciences (Approval Number: 70-25-0001), and was conducted in accordance with the guidelines of the Declaration of Helsinki. We retrospectively enrolled 63 patients diagnosed with mRCC who received first-line IO–IO therapy between September 2018 and August 2020 with a minimum potential follow-up of 5 years at Nagoya City University Hospital and seven affiliated institutions. mRCC diagnosis was confirmed by experienced pathologists through histological analysis. The indication for IO–IO therapy was determined based on discussions among urologists and consideration of patients’ characteristics, including through use of the International Metastatic Renal Cell Carcinoma Database Consortium (IMDC) risk classification [16], and was finalized in agreement with the patient. All patients received IO–IO inducting therapy as the first line of treatment, encompassing nivolumab (240 mg) and ipilimumab (1 mg/kg) administered every 3 weeks for four cycles. Patients without disease progression or unacceptable toxicity proceeded to nivolumab maintenance therapy, which was administered every 2 or 4 weeks. The treatment was continued until disease progression, unacceptable toxicity, the patient’s preference was to stop, or until sufficient clinical benefit was reached. Baseline and on-treatment assessments included medical history, demographic and physical examinations, Karnofsky Performance Status, age-unadjusted Charlson Comorbidity Index [17], and blood and urine tests, all conducted at the discretion of the attending physician.

### 2.2. Outcomes

PFS was defined as the interval between the treatment initiation date and the date of disease progression or death. PFS2 was defined as the time from the initiation of first-line treatment to the date of second disease progression or death. OS was defined as the interval between the IO–IO initiation date and the date of death. Patients lost to follow-up were censored at the date of last contact. Treatment response after initiating IO–IO was assessed according to the Response Evaluation Criteria in Solid Tumors version 1.1 [18], and categorized as complete response (CR), partial response (PR), stable disease (SD), or progressive disease (PD). Radiographic assessments were performed following routine clinical practice at the discretion of the attending physician, typically every 8–12 weeks, although the exact timing was not standardized across institutions. PF–TF survival was defined as a composite binary outcome indicating the absence of disease progression or any cancer-directed therapy at the 5-year landmark after treatment initiation (PF–TF group). Patients who did not achieve PF–TF at 5 years were classified as the non-PF–TF group. TF status was defined as the absence of any systemic therapy or local cancer-directed treatment at the 5-year landmark.

Adverse events were graded according to the Common Terminology Criteria for Adverse Events, version 5.0. Immune-related adverse events (irAEs) were defined as adverse events considered by the attending physicians to be immune-related and were evaluated for exploratory analyses. Blood test values were obtained at baseline and before the second cycle, at the end or discontinuation of IO–IO induction therapy, and at the 12-month follow-up. The systemic immune inflammation index was calculated as platelet count × neutrophil count/lymphocyte count [19].

### 2.3. Statistical Analysis

Fisher’s exact test (for stratification factors) and the Mann–Whitney U test (for continuous variables) were used to compare patients’ characteristics and laboratory data. In addition, the interquartile range (IQR) was used to report continuous variables. PFS, PFS2, and OS were stratified using the Kaplan–Meier method and analyzed using the log-rank test. All the reported *p*-values were two-sided, with the statistical significance set at *p* < 0.05. Missing laboratory values were not imputed. Statistical analyses were performed using the EZR software (version 1.66; Saitama Medical Center, Jichi Medical University, Saitama, Japan) [20]. Patient treatment profiles at the 12-month and 5-year landmarks after treatment initiation were visualized using Sankey diagrams, which were generated with SankeyMATIC (https://sankeymatic.com/; accessed on 20 December 2025). For the 12-month landmark analysis, only patients who were alive and evaluable at 12 months were included, in accordance with landmark methodology.

## 3. Results

### 3.1. Patient Characteristics

Table 1 summarizes the baseline characteristics of the patients prior to the initiation of IO–IO therapy. The cohort consisted of patients with intermediate- or poor-risk disease according to the IMDC classification, with no favorable-risk patients included. Clear cell renal cell carcinoma was the most common histological subtype, and the lung was the most common site of metastasis, followed by bone and lymph node involvement. A total of 13 patients (21%) had undergone nephrectomy before the initial visit, reflecting the treatment for localized disease. Among 50 patients (78%) who had not undergone nephrectomy at baseline, 8 underwent deferred cytoreductive nephrectomy (CN). The median follow-up duration was 24.3 months (IQR, 5.0–65.0 months). The details of the surgical procedures and subsequent treatment patterns are summarized in Tables S1 and S2.

### 3.2. Antitumor Efficacy

**Figure 1:**
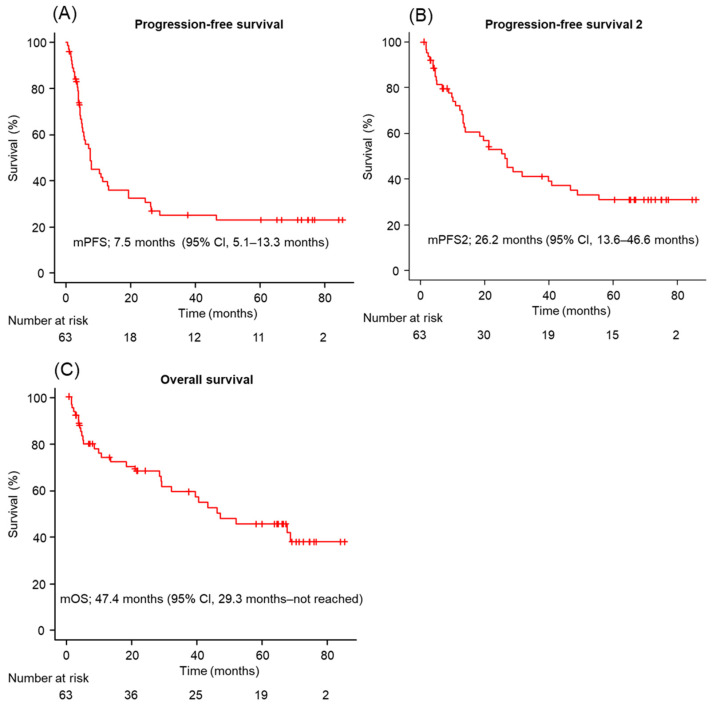
Survival outcomes. (**A**–**C**) Kaplan–Meier survival curves for (**A**) progression-free survival (n = 63), (**B**) progression-free survival 2 (n = 63), and overall survival (n = 63) in patients. (**A**–**C**) *p*-values were calculated using the log-rank test. Abbreviations: mPFS: median progression free survival; mPFS2: median progression-free survival 2; mOS: median overall survival; and 95% CI: 95% confidence interval.

The median PFS, PFS2, and OS for the entire cohort were 7.5 months (95% confidence interval [CI]; 5.1–13.3), 26.2 months (95% CI; 13.6–46.6), and 47.4 months (95% CI; 29.3–not reached), respectively (Figure 1). The landmark PFS rates at 12, 24, 36, 48, and 60 months were 39.6%, 32.4%, 25.0%, 23.0%, and 23.0%, respectively. The corresponding OS rates were 74.2%, 68.0%, 58.9%, 48.0%, and 46.4%. The best overall response during IO–IO induction therapy was CR in 3 patients (5%), PR in 23 (37%), SD in 19 (30%), and PD in 16 (25%). The objective response rate (ORR) was 43%, and the disease control rate (DCR) was 74% (Table 2).

**Figure 2:**
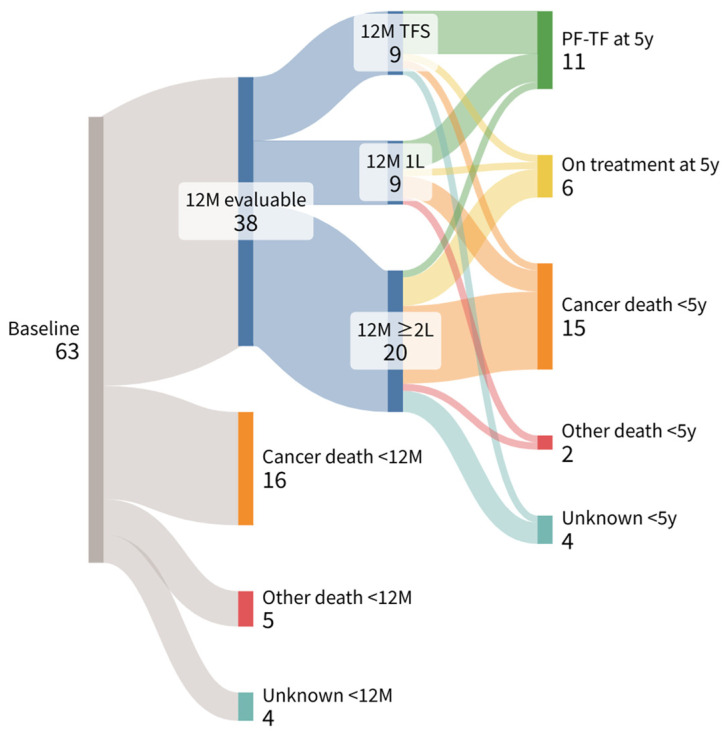
Patient treatment profiles at 12-month and 5-year landmark after treatment initiation visualized using a Sankey diagram. Abbreviations: 12M: 12-month landmark; TF: treatment-free; 1L: first-line therapy; ≥2L: second- or later-line therapy: PF–TF: progression-free and treatment-free; and 5y: 5-year landmark.

Patient treatment status at 12 months and subsequent clinical outcomes at the 5-year landmark are shown in Figure 2 using Sankey diagrams. At the 12-month landmark, 38 patients (60%) were alive and evaluable, 16 (25%) had died of cancer, 5 (8%) had died of other causes, and 4 (7%) were lost to follow-up. At the 5-year landmark, among evaluable patients, 11 (17%) achieved PF–TF, 6 (10%) were receiving ongoing therapy, 15 (24%) had died of cancer, 2 (3%) had died of other causes, and 4 (6%) had unknown outcomes. Notably, patients who ultimately achieved PF–TF survival at 5 years were distributed across different treatment statuses at the 12-month landmark, including being treatment-free and receiving ongoing first- or later-line therapies.

### 3.3. Safety

Table 3 shows the summary of irAEs. Overall, 43 patients (68%) experienced irAEs of any grade. Twenty-four patients (38%) experienced grade ≥3 irAEs, twenty-nine patients (46%) required initiation of systemic steroid therapy, and six patients (10%) received steroid pulse therapy. Eighteen patients (29%) discontinued treatment due to irAEs. Three patients (5%) died due to irAEs. The most frequently reported irAEs were a skin rash or pruritus (17%), colitis or diarrhea (13%), interstitial pneumonia (11%), and adrenal insufficiency (11%). Among grade ≥3 irAEs, interstitial pneumonia and adrenal insufficiency were the most common, each occurring in 8% of patients (Table S3).

### 3.4. Comparison of Clinical Characteristics and Outcomes Between PF–TF and Non-PF–TF Patients

Table 4 compares baseline characteristics, laboratory findings, and irAEs between the PF–TF and non-PF–TF groups. Baseline demographic and clinical characteristics, including age, IMDC risk classification and pretreatment laboratory parameters, were comparable between the two groups. In contrast, metastatic patterns differed: no patients in the PF–TF group had bone metastases, whereas lymph node (LN) metastases were more frequent (*p* = 0.01 and *p* < 0.01, respectively). Deferred CN was more common in the PF–TF group, whereas a higher proportion of patients in the non-PF–TF group underwent upfront CN or did not undergo nephrectomy during follow-up. All patients in the PF–TF group experienced at least one irAE, with a significantly higher incidence compared to the non-PF–TF group (*p* = 0.01). However, the incidence of grade ≥3 irAEs and systemic corticosteroid use did not differ significantly between groups. No patients in the PF–TF group required steroid pulse therapy. The median time to the first irAE was significantly longer in the PF–TF group than in the non-PF–TF group (*p* < 0.01).

Patients in the PF–TF group were more likely to complete IO–IO induction therapy (*p* = 0.04) and had longer duration of IO–IO therapy than those in the non-PF–TF group (*p* = 0.01; Table S4). The median duration of IO–IO therapy in the PF–TF group was 22 weeks (IQR, 14–70 weeks). The reasons for treatment discontinuation in this group were the achievement of sufficient clinical benefit in five patients, adverse events in five patients, and disease progression in one patient. Among the patients who discontinued treatment due to sufficient clinical benefit, the median treatment duration was 76 weeks. Notably, all patients who discontinued treatment due to adverse events did so within 18 weeks of initiation. In contrast, the median duration in the non-PF–TF group was 12 weeks (IQR, 6–22 weeks).

Among patients evaluable at the 12-month landmark, no significant differences were observed between the PF–TF and non-PF–TF groups in longitudinal laboratory parameters, including eosinophil counts at the time of irAE onset (Table S5).

## 4. Discussion

To our knowledge, this is one of the first real-world studies to report long-term outcomes with a minimum follow-up of 5 years in patients with mRCC treated with IO–IO, with a particular focus on PF–TF survival at the 5-year landmark. Notably, despite including patients who would have been excluded from the CheckMate 214 trial—such as those with brain metastases or non-clear cell histology [3]—our cohort demonstrated outcomes comparable in terms of PFS2, OS, ORR, and DCR. Although these findings appear to contrast with reports from large clinical trials suggesting poorer outcomes in patients who are ineligible for immunotherapy trials [21], they are largely consistent with other real-world data [15,22]. In this analysis, 11 of 63 patients (17%) treated with IO–IO achieved PF–TF survival, indicating that a subset of patients can experience sustained clinical benefit even after discontinuation of immunotherapy. These findings support the existence of durable PF–TF survival and a long-lasting benefit of immune checkpoint blockade in real-world clinical practice. The Sankey diagram shows that patients who ultimately achieved PF–TF survival status at the 5-year landmark were distributed across all treatment phases at the 12-month time point, including treatment-free, ongoing first-line therapy, and second-line or later therapy. This finding is consistent with prior reports suggesting that durable disease control can be observed even after early treatment discontinuation and that delayed or atypical response patterns may occur independently of the initial treatment course [23].

One of the key findings of this study is that baseline factors—including age, IMDC risk classification, and peripheral blood biomarkers—were not predictive of PF–TF achievement. Even when longitudinal laboratory data obtained during induction therapy and up to the 12-month time point were analyzed, no significant differences were observed between the two groups. In contrast, differences were observed with respect to metastatic patterns. Specifically, the PF–TF group was characterized by the absence of bone metastases, a higher frequency of LN metastases, the occurrence and time to onset of irAEs, and more frequent deferred CN. Bone metastases may reflect a high systemic tumor burden, which can limit effective immune activation by ICI and thereby hinder the establishment of durable immune-mediated disease control [24,25]. Lymph node-only metastasis was observed in both groups, and most patients with lymph node involvement had multiple metastatic sites, suggesting that this association cannot be fully explained by differences in tumor burden. Recent reports have highlighted the crucial role of tumor-draining lymph nodes (TDLNs) in mediating responses to ICI [26,27]. Given that TDLNs and metastatic LNs are biologically distinct entities, the higher frequency of LN metastases in the PF–TF group should be interpreted with caution. Nevertheless, considering the fundamental role of LNs in T-cell priming and immune activation, nodal involvement may reflect an immunological milieu conducive to durable systemic disease control. Deferred CN was more frequently observed in the PF–TF group, whereas upfront CN or no nephrectomy was more common in the non-PF–TF group. Considering emerging evidence favoring deferred CN, rather than upfront CN, in the era of ICI [28,29,30,31,32], including recent reports suggesting a potential benefit in selected patients [32], this observation suggest a potential association between deferred CN and favorable long-term outcomes in selected patients. However, given the retrospective nature of this study and potential selection bias, this finding should be interpreted cautiously, as deferred CN may reflect a strategy applied to selected responders rather than an independent determinant of long-term PF–TF survival. Biomarkers associated with long-term outcomes in mRCC have recently begun to emerge particularly in the context of TKI monotherapy [33]. However, such biomarker-based insights remain relatively limited in ICI-based combination therapies.

With respect to adverse events, all patients in the PF–TF group experienced at least one irAE, whereas the incidence of grade ≥3 irAEs did not differ significantly between the two groups. These observations are consistent with previous reports demonstrating an association between the occurrence of irAEs and favorable clinical outcomes, as well as the notion that treatment discontinuation due to severe irAEs does not necessarily preclude the possibility of long-term survival [19,34,35,36]. Together, these findings support an association between PF–TF and the occurrence of irAEs. Although the rate of corticosteroid initiation was comparable between groups, notably, no patients in the PF–TF group required high-dose corticosteroid pulse therapy, despite the absence of a statistically significant difference. While direct evidence in mRCC remains limited, large-scale studies in other tumor types have demonstrated that peak corticosteroid dose administered for irAEs management adversely affects overall survival during immunotherapy, whereas cumulative corticosteroid exposure does not correlate with survival outcomes [37,38]. The present findings are concordant with this concept. Notably, the time to first irAEs was significantly longer in the PF–TF group than in the non-PF–TF group. In RCC, limited evidence links early-onset irAEs to favorable outcomes [39]; however, this relationship is based on comparisons between patients with and without irAEs rather than direct evaluation of early versus late onset. In contrast, studies across various malignancies directly comparing early and late onset suggest that delayed irAEs may be associated with improved survival [40,41], which is consistent with our findings. However, this observation should be interpreted cautiously. In addition, although we have previously reported an association between irAEs development and eosinophil elevation [42,43], this study did not demonstrate a dose-dependent relationship between eosinophil counts and long-term outcomes in the present cohort, suggesting a limited role in predicting durable PF–TF survival. Collectively, these findings suggest that irAEs severity itself or treatment discontinuation does not necessarily compromise long-term clinical outcomes, whereas excessive immunosuppression—particularly high-dose corticosteroid pulse therapy—may adversely affect durable antitumor benefit by attenuating sustained immune activation. However, as patients in the PF–TF group tended to receive longer IO–IO therapy, this may have increased the likelihood of developing irAEs. This association should be interpreted with caution. Nevertheless, the high incidence of severe irAEs and occurrence of irAE-related deaths underscore the clinically significant risks associated with IO–IO therapy. To mitigate these risks and optimize patient safety, careful patient selection, thorough informed consent, and early intervention at the onset of irAEs are essential.

Determining the optimal timing for treatment discontinuation in patients who respond to IO–IO remains challenging; no international guidelines currently define when ICI therapy should be discontinued. Alexander et al. analyzed patients who had received IO–IO or nivolumab monotherapy for at least 21 months and reported that durable responses to immunotherapy may not be determined solely by the IMDC risk classification [44]. They further concluded that the planned discontinuation of therapy at approximately 24 months after treatment initiation was unlikely to compromise favorable clinical outcomes. In this study, the median duration of IO–IO therapy among patients who discontinued treatment due to sufficient clinical benefit was 76 weeks, and only two patients in the PF–TF group received ICI for more than 24 months. Notably, approximately half of the PF–TF group discontinued treatment early because of irAEs and did not receive further immunotherapy thereafter. The observation that long-term responses were achieved even in these early discontinuation cases suggests that the clinical efficacy of ICIs may not be solely dependent on the cumulative dose administered. In this regard, Georgy et al. reported in head and neck cancer that low-dose nivolumab was sufficient to maintain PD–1 receptor saturation and exert adequate antitumor activity [45]. Taken together, these findings suggest that in IO–IO therapy for mRCC, achieving and maintaining an immunological activation exceeding a certain threshold during the induction phase may be sufficient to induce a durable PF–TF state, irrespective of long-term treatment continuation. These distinctive therapeutic features of IO–IO may have implications for treatment selection among the available first-line options, including IO–TKI regimens, which are characterized by rapid tumor shrinkage and early disease control [46]. These findings suggest distinct therapeutic profiles for different ICI-based combination strategies, with no single approach currently providing a comprehensive solution for all patients. Among these approaches, IO–IO therapy may offer a unique opportunity for durable PF–TF survival in selected patients.

This study has several limitations. First, this was a retrospective study with a relatively small sample size, which may limit the generalizability of our findings, warranting cautious interpretation. In addition, the limited number of the PF–TF group and the presence of complete separation in key variables precluded multivariable analysis and decreased the robustness of statistical inference. Therefore, the observed associations should be interpreted as descriptive and hypothesis-generating rather than definitive. Second, as PF–TF was assessed as a 5-year landmark-based outcome, it may be subject to survivorship bias. This approach may also be affected by informative censoring and potential misclassification due to missing follow-up data and heterogeneous treatment trajectories. Notably, the relatively short median follow-up duration reflects early events and censoring rather than insufficient observation time. Third, treatment decisions, including treatment discontinuation, steroid use for irAEs, and subsequent therapies, were not standardized and were made at the discretion of the treating physicians, which may have influenced clinical outcomes and introduced potential bias. In addition, this cohort consisted predominantly of Japanese patients, which may limit the generalizability of our findings to other populations. Despite these limitations, few real-world studies have reported long-term outcomes with a minimum follow-up of 5 years focusing on sustained response, and our findings provide clinically relevant insights. Future prospective randomized studies are warranted to validate these results.

## 5. Conclusions

This real-world study with a minimum follow-up of 5 years demonstrated the sustained efficacy of IO–IO in patients with mRCC. At 5 years, 17% of patients remained free from both disease progression and subsequent therapy. Durable PF–TF survival was not predicted by baseline risk or blood-based biomarkers, but was associated with metastatic patterns and the development of irAEs, and was observed even after early treatment discontinuation. These findings suggest that IO–IO and IO–TKI regimens fulfill distinct therapeutic roles in the management of mRCC. IO–IO represents a treatment strategy that may achieve sustained immune-mediated disease control and treatment-free survival through effective immune activation during the induction phase. Therefore, an optimal treatment strategy for mRCC should be based on an understanding of the differing characteristics of these regimens, taking into account individual patient profiles and treatment goals.

## Figures and Tables

**Table 1:** Patients’ characteristics.

Number of Patients 63 (100%)	
Gender (%): Male	46 (73)
Age (year): Median (IQR)	70.0 (63.5–74.5)
BMI (kg/m^2^): Median (IQR)	21.4 (19.5–24.6)
Karnofsky Performance Status: ≥80	43 (68)
Comorbidity	
Cardiovascular disease	13 (21)
Diabetes mellitus	12 (19)
Other malignancy	6 (10)
Autoimmune disease	1 (2)
Charlson Comorbidity Index (%)	
0	29 (46)
1–2	27 (43)
≥3	7 (11)
IMDC risk classification (%)	
Intermediate	32 (51)
Poor	31 (49)
Histology * (%)	
Clear cell	48 (76)
Other	6 (10)
Unknown	9 (14)
Number of metastatic sites (%)	
1	32 (51)
2	20 (32)
≥3	11 (17)
Site of metastasis (%)	
Lung: Yes	41 (65)
Bone: Yes	21 (33)
Liver: Yes	12 (19)
Brain: Yes	4 (6)
Lymph node: Yes	16 (25)
History of nephrectomy (%)	
Prior nephrectomy (for localized disease)	14 (22)
Nephrectomy–naive at baseline	
Upfront CN	12 (19)
Deferred CN	8 (13)
No CN	29 (46)

* Other histologies include non-clear cell subtypes (e.g., chromophobe RCC) and cases with ambiguous or non-definitive classification (e.g., “clear cell-like”). Unknown indicates cases in which the histological subtype could not be determined, including biopsy-only diagnoses. Sarcomatoid features were included within the corresponding histological subtype. Abbreviations: IQR: interquartile range; BMI: body mass index; IMDC: International Metastatic Renal Cell Carcinoma Database Consortium; and CN: Cytoreductive Nephrectomy.

**Table 2:** Treatment response.

Number of Patients 63 (100%)	
Best response	
CR	3 (5)
PR	23 (37)
SD	19 (30)
PD	16 (25)
Not Assessed	2 (3)
Objective response	26 (43)
Disease control	45 (72)

Abbreviations: CR: complete response; PR: partial response; SD: stable disease; and PD: progressive disease.

**Table 3:** Summary of irAEs.

Number of Patients 63 (100%)	
Any grade	43 (68)
Grade3 or more	24 (38)
Corticosteroid therapy *	29 (46)
Steroid pulse therapy *	6 (10)
Time to first irAE * (week): Median (IQR)	6.0 (2.9–12.4)
Discontinuation due to irAEs	18 (29)
Death due to irAEs	3 (5)

* Proportions and time were calculated in patients who experienced at least one irAE. Abbreviations: irAEs: immune-related adverse events; IQR: interquartile range.

**Table 4:** Comparison of baseline patient characteristics, irAEs and laboratory findings.

	PF–TF Group (n = 11)	Non-PF–TF Group (n = 52)	*p*-Value
Gender (%): Male	6 (55)	40 (77)	0.15
Age (year): Median (IQR)	73 (68–79.5)	69.5 (60.8–74.0)	0.18
BMI (kg/m^2^): Median (IQR)	22.8 (21.1–25.2)	21.3 (19.1–24.6)	0.40
Karnofsky Performance Status: ≥80	8 (73)	35 (67)	1.00
Comorbidities (%)			
Cardiovascular disease	2 (18)	11 (21)	1.00
Diabetes mellitus	2 (18)	10 (19)	1.00
Other malignancy	1 (9)	5 (10)	1.00
Autoimmune disease	0 (0)	1 (2)	1.00
Charlson Comorbidity Index (%)			0.52
0	6 (55)	23 (44)	
1–2	5 (45)	22 (43)	
≥3	0 (0)	7 (13)	
IMDC risk classification (%)			1.00
Intermediate	6 (55)	26 (50)	
Poor	5 (45)	26 (50)	
Histology *^1^			1.00
Clear cell	9 (82)	39 (75)	
Other	1 (9)	5 (10)	
Unknown	1 (9)	8 (15)	
Number of metastatic sites (%)			0.74
1	7 (64)	25 (48)	
2	3 (27)	17 (33)	
≥3	1 (9)	10 (19)	
Site of metastasis (%)			
Lung	6 (55)	35 (67)	0.49
Bone	0 (0)	21 (40)	**0.01**
Liver	2 (18)	10 (19)	1.00
Brain	0 (0)	4 (8)	1.00
Lymph node	7 (64)	9 (17)	**<0.01**
History of nephrectomy (%)			**<0.01**
Prior nephrectomy (for localized disease)	4 (36)	11 (21)	
Upfront CN	0 (0)	12 (24)	
Deferred CN	6 (55)	2 (4)	
No CN	1 (9)	28 (54)	
irAEs (%)			
Any Grade	11 (100)	32 (62)	**0.01**
Grade 3 or more	3 (27)	21 (40)	0.51
Corticosteroid therapy *^2^	7 (64)	22 (68)	0.73
Steroid pulse therapy *^2^	0 (0)	6 (19)	0.31
Time to first irAE (week): Median (IQR) *^2^	12.9 (7.4–27.1)	4.5 (2.0–8.3)	**<0.01**
Blood data before initiation			
Hb (g/dL): Median (IQR)	12.0 (8.56–12.78)	11.4 (9.7–12.7)	0.93
SII (IQR)	896 (669–1239)	937 (674–1598)	0.48
ALC (10^9^/L): Median (IQR)	1.30 (0.97–1.61)	1.20 (0.93–1.80)	0.55
AEC (10^9^/L): Median (IQR)	0.15 (0.11–0.23)	0.15 (0.11–0.24)	0.96
CRP (mg/L): Median (IQR)	0.34 (0.16–1.97)	1.92 (0.39–7.27)	0.12
Corrected Ca (mg/dL): Median (IQR)	9.6 (9.3–9.9)	9.8 (9.3–10.3)	0.49

Bold values indicate statistical significance (*p* < 0.05). *^1^ Statistical analysis was performed only for patients with clear cell or other histological subtypes, excluding those with unknown histology. *^2^ Proportions and time were calculated in patients who experienced at least one irAE. Abbreviations: PF–TF: progression-free and treatment-free; IQR: interquartile range; BMI: body mass index; IMDC: International Metastatic Renal Cell Carcinoma Database Consortium; CN: Cytoreductive Nephrectomy; irAEs: immune-related adverse events; Hb: hemoglobin; SII: systemic immune inflammation index; ALC: absolute lymphocyte count; AEC: absolute eosinophil count; CRP: C-reactive protein; and Ca: calcium.

## Data Availability

Materials and raw data may be given after request made to the corresponding author.

## Supplementary Materials

The following supporting information can be downloaded at: https://www.mdpi.com/article/10.3390/cancers18081315/s1. Table S1: Details of the surgical procedures; Table S2: Details of second-line and third-line regimens; Table S3: Details of the irAEs profiles; Table S4: Treatment Exposure and Discontinuation Patterns; and Table S5: Landmark Blood test data.
