# Supplementary material for: Durable Progression-Free and Treatment-Free Survival After Nivolumab Plus Ipilimumab Therapy in Metastatic Renal Cell Carcinoma: A Real-World Study with a 5-Year Minimum Follow-Up
Source: Cancers (Basel). 2026 Apr 21;18(8):1315. doi: 10.3390/cancers18081315 (PMC13115136; doi:10.3390/cancers18081315)
Supplement: Supplementary file 1 [file cancers-18-01315-s001.zip › cancers-4217479-supplementary.pdf]

Supplementary Materials: Durable Progression-Free and Treatment-Free Survival After Nivolumab Plus Ipilimumab Therapy in Metastatic Renal Cell Carcinoma: A Real-World Study with a 5-Year Minimum Follow-Up

Hiroaki Ikoma, Shuzo Hamamoto, Yoshihiko Tasaki, Misato Tomita, Kengo Kawase, Hiroko Suzuki, Yusuke Noda, Masayuki Usami, Yohei Tsubouchi, Ryuga Kato, Takuya Sakata, Yoshihisa Mimura, Toshiharu Morikawa, Takashi Nagai, Rei Unno, Toshiki Etani, Taku Naiki, Yosuke Sugiyama and Takahiro Yasui

Table S1. Details of the surgical procedures

History of nephrectomy (%)	ORN	LRN	RARN	Unknown	No CN	Total
Prior nephrectomy (for localized disease)	4 (6)	9 (14)	0 (0)	1 (2)	0 (0)	14 (22)
Upfront CN	3 (5)	9 (14)	0 (0)	0 (0)	0 (0)	12 (19)
Deferred CN	6 (10)	1 (2)	1 (2)	0 (0)	0 (0)	8 (13)
No CN	0 (0)	0 (0)	0 (0)	0 (0)	29 (46)	29 (46)
Total	13 (21)	19 (30)	1 (2)	1 (2)	29 (46)	63 (100)

Abbreviations: ORN, open radical nephrectomy; LRN, laparoscopic radical nephrectomy; RARN, robot-assisted radical nephrectomy; CN, Cytoreductive Nephrectomy.

Table S2. Details of second-line and third-line regimens

Number of patients 63	
Reached 2nd therapies (n=32, 100%)	n, (%)
Axitinib	10 (31)
Cabozantinib	5 (16)
Sunitinib	5 (16)
Pazopanib	5 (16)
Sorafenib	1 (3)
Everolimus	1 (3)
Other	5 (16)
Reached 3rd therapies (n=16, 100%)	n, (%)
Cabozantinib	6 (38)
Axitinib	4 (25)

Everolimus	3 (19)
Pazopanib	2 (12)
Nivolumab	1 (6)

Table S3. Details of the AEs profiles

Number of patients 63 (100%)		
Profile of AEs, n, (%)	Any Grade	Grade3 or more
Skin rash or pruritus	11(17)	1 (2)
Colitis or diarrhea	8(13)	3 (5)
Interstitial pneumonia	7(11)	5 (8)
Adrenal insufficiency	7(11)	5 (8)
Hypothyroidism	6(10)	0 (0)
Destructive thyroiditis	5(8)	1 (2)
Hypophysitis	4(6)	3 (5)
Hepatic dysfunction	4(6)	2 (3)
Type1 diabetes mellitus	3(5)	3 (5)
Neuropathy	3(5)	2 (3)
Myositis or myocarditis	2(3)	1 (2)
Renal dysfunction	2(3)	1 (2)
Encephalitis	1(2)	1 (2
Negative myoclonus	1(2)	1 (2)

Abbreviations: AEs, adverse events

Table S4. Treatment Exposure and Discontinuation Patterns

	PF–TF group (n=11)	Non–PF–TF group (n=52)	p-value
Completed 4 cycles (%)	10 (91)	31 (52)	0.04
Duration of IO–IO therapy (week): Median (IQR)	22 (14-70)	12 (6-22)	0.01
Discontinuation reason* (%)			
Clinical benefit	5 (36)	1 (2)	
AEs	5 (46)	12 (23)	
PD/death	1 (9)	30 (58)	
Patient preference	0 (0)	6 (11)	
Others	0 (9)	3 (6)	

P-values were calculated for comparisons of induction completion and treatment duration. Discontinuation reasons are presented descriptively.

Abbreviations: AEs, adverse events; PD, Progressive disease

Table S5. Landmark Blood test data

Blood data: Median (IQR)	PF–TF group (n=11)	Non–PF–TF group*[1] (n=27)	p-value
At baseline			
Hb (g/dL)	12.0 (8.56-12.78)	11.4 (9.7-12.7)	0.93
SII	896 (669-1239)	937 (674-1598)	0.48
ALC (10^9/L)	1.30 (0.97-1.61)	1.20 (0.83-1.80)	0.55
AEC (10^9/L)	0.15 (0.11-0.23)	0.15 (0.11-0.24)	0.96
CRP (mg/L)	0.34 (0.16-1.97)	1.44 (0.34-2.73)	0.12
Corrected Ca (mg/dL)	9.6 (9.3-9.9)	9.7 (9.3-10.1)	0.83
Before the second cycle			
Hb (g/dL)	11.7 (9.38-13.1)	11.6 (10.5-13.1)	0.44
SII	1074 (520-1177)	782 (507-1001)	0.41
ALC (10^9/L)	1.50 (0.99-1.77)	1.61 (1.09-2.42)	0.32
AEC (10^9/L)	0.24 (0.22-0.53)	0.20 (0.16-0.40)	0.36
CRP (mg/L)	0.60 (0.47-1.53)	0.89 (0.24-2.65)	0.65
Corrected Ca (mg/dL)	9.6 (9.2-9.9)	9.6 (9.2-10.3)	0.77
At the end or discontinuation of the four-cycle			
Hb (g/dL)	12.1 (10.8-13.4)	12.2 (11.1-13.1)	0.88
SII	792 (741-1318)	780 (613-1704)	0.99
ALC (10^9/L)	1.69 (1.37-1.86)	1.46 (1.15-2.29)	0.99
AEC (10^9/L)	0.20 (0.15-0.61)	0.24 (0.13-0.44)	0.69
CRP (mg/L)	0.16 (0.11-0.92)	0.45 (0.12-1.85)	0.35
Corrected Ca (mg/dL)	9.5 (9.4-9.8)	9.5 (9.3-9.9)	1.00
At the 12-month follow-up			
Hb (g/dL)	12.1 (11.5-13.0)	13.5 (12.2-14.3)	0.18
SII	737 (459-1248)	660 (318-948)	0.48
ALC (10^9/L)	1.60 (1.06-2.68)	1.50 (0.93-1.83)	0.29
AEC (10^9/L)	0.20 (0.17-0.28)	0.24 (0.16-0.40)	0.40
AEC at irAEs onset*[2]	0.32 (0.26-0.47)	0.30 (1.14-0.52)	0.49

*[1] Patients who were alive at 12–months landmark and had available blood test results at each landmark.

*[2] Blood parameters were analyzed in patients who experienced at least one irAE.

Abbreviations: PF–TF, progression-free and treatment-free survival; IQR, Interquartile range; Hb, hemoglobin; SII, systemic immune-inflammation index; ALC, absolute lymphocyte count; AEC, absolute eosinophil count; CRP, C-reactive protein; Ca, calcium; irAEs, immune-related adverse events
